# Profiles of older adults according to their life and food-related life satisfaction during the COVID-19 pandemic: the importance of the social environment

**DOI:** 10.3389/fpubh.2023.1165256

**Published:** 2023-08-17

**Authors:** Berta Schnettler, Germán Lobos, Ligia Orellana, Cristian Adasme-Berríos, María Lapo, Katherine Beroíza

**Affiliations:** ^1^Facultad de Ciencias Agropecuarias y Medioambiente, Universidad de La Frontera, Temuco, Chile; ^2^Scientific and Technological Bioresource Nucleus (BIOREN-UFRO), Universidad de La Frontera, Temuco, Chile; ^3^Centro de Excelencia en Psicología Económica y del Consumo, Universidad de La Frontera, Temuco, Chile; ^4^Universidad Católica de Santiago de Guayaquil, Guayaquil, Ecuador; ^5^Facultad de Economía y Negocios, Universidad de Talca, Talca, Chile; ^6^Núcleo de Ciencias Sociales y Humanidades, Universidad de La Frontera, Temuco, Chile; ^7^Departamento de Psicología, Universidad de La Frontera, Temuco, Chile; ^8^Departamento de Economía y Administración, Universidad Católica del Maule, Talca, Chile

**Keywords:** older adults, life satisfaction, food-related life satisfaction, latent profile analysis, COVID-19

## Abstract

**Introduction:**

Older adults are a highly heterogeneous population, as individuals of the same age can show considerable variations in personal characteristics and living conditions. Risk and protective factors for older adults' subjective wellbeing during the COVID-19 pandemic can be explored by examining how life satisfaction, food-related life satisfaction, and associated variables coexist among these individuals. On this basis, this study aimed to identify older adult profiles based on their levels of life and food-related life satisfaction; to characterize these profiles by diet quality, social support, financial wellbeing, and sociodemographic characteristics; and to identify variables associated with higher life and food-related life satisfaction.

**Methods:**

The sample included 1,371 institutionalized and non-institutionalized individuals over the age of 60, from four cities in Chile. Participants answered a survey, either online or face to face, with questions about life and food-related life satisfaction, perceived social support from family, friends, and others, food quality, financial wellbeing/distress, sociodemographic characteristics, and prior COVID-19 infection.

**Results:**

Using a latent profile analysis, we identified three profiles of older adults: Profile 1: Unsatisfied with their life, somewhat satisfied with their food-related life (5.40%); Profile 2: Somewhat satisfied with their life, satisfied with their food-related life (65.06%); Profile 3: Extremely satisfied with their life and food-related life (29.54%). Profiles differed by residence (institutionalized vs. independent), age, marital status, social support, financial wellbeing, COVID-19 infection, and city of residence.

**Discussion:**

The patterns of association between life and food-related satisfaction and related variables indicate conditions of vulnerability and protection related to living conditions, the social dimensions of food consumption, and social support. These results underscore the need for identifying groups of older adults based on diverse characteristics and conditions outside of chronological age.

## 1. Introduction

Around the world, 703 million people are 65 years or older, and this population is expected to increase to 1.5 billion by 2050 (1). As both life expectancy and the population of older people increase worldwide, improving older adults' wellbeing is of utmost importance (2). Wellbeing is an important indicator of successful or active aging and longevity (3, 4), but there is no one-size-fits-all intervention to foster wellbeing in older adults because they constitute a very heterogeneous group [e.g., (4–7)]. Indeed, the World Health Organization (5) highlights that chronological age is not an indicator of changes that come with aging. Older adults of the same age show considerable variations in health status, degree of disability and involvement in everyday and societal activities, and autonomy, among other variables (8).

One of the most relevant constructs to measure individuals' subjective wellbeing is life satisfaction (9), defined as the assessment that people make of their overall life or specific domains of their life (10). Among the latter, the food domain has received growing interest in the literature about older adults' wellbeing. In this line, food-related life satisfaction is an assessment of food-related wellbeing (11). Studies in Europe, Asia, and Latin America have demonstrated a positive link between life and food-related life satisfaction in older adults (2, 11–18). Moreover, higher food-related life satisfaction in older adults has been associated with healthier eating habits (2, 7, 13, 14, 17, 19), the social and hedonic dimensions of food (2, 7, 15, 17), and some food attributes such as healthfulness, variety (2), and safety (14). Hence, we expect that older adults show diverse patterns of life and food-related life satisfaction and that these patterns coexist with other factors and conditions associated with wellbeing.

A significant threat to older adults' wellbeing has been the COVID-19 pandemic declared in March 2020. This pandemic disrupted the daily life of many populations as it posed several health threats and required highly restricting measures to curb the infection. Studies during the pandemic have shown that within older adult populations there are subgroups with higher vulnerability such as women, single people, ethnic minorities, people with pre-existing physical and mental illness, and people of lower socioeconomic status (4, 20). These studies also suggest that pronounced negative effects may be expected among individuals with fewer social (e.g., family, friends, and support networks), psychological (e.g., emotional stability and sense of control), and socioeconomic (e.g., education, income, financial wellbeing) resources (4, 20). Among these factors, financial wellbeing is relevant as a component of a broader range of subjective dimensions, and it refers to a multidimensional construct that includes both objective (e.g., income, availability of cash, net worth, or income stability) and subjective measures (e.g., financial satisfaction, financial behaviors, and financial perceptions) of individuals' financial situation (21).

Older adults have also faced other challenges during the pandemic. Older populations were required to abstain from social and pleasurable activities, such as meeting with friends and family, and from performing meaningful and socially valued roles such as grandparenting (22, 23). In consequence, many older adults had restricted access to sources of support and caregiving (20). Furthermore, prolonged lockdowns aimed to prevent infection were associated with several risk factors for older adults' mental health, such as increased feelings of loneliness (4, 20). The literature from developed countries reports that older adults have shown high resilience and stable wellbeing during the early stages of the pandemic (4, 23). Research on older adults' wellbeing in the later stages of the pandemic (i.e., late 2020 and onward) is still scarce, but studies so far suggest that these later stages may have compromised the wellbeing of this population more than the earlier stages (4). Moreover, this latter research has stressed the heterogeneity among older adults during the pandemic (23), which translates into different reactions and patterns of vulnerability to COVID-19 (22, 24).

A variable-centered approach, that is, an approach focused on examining relations among variables (e.g., correlations, regression), may not properly address these complex interrelations between factors from different life domains and life and food-related life satisfaction in older adults. Research on life satisfaction traditionally reported a “U shape” trajectory, as it decreased in middle age and increased again in older age, but there is evidence that this pattern is not consistent (25–27). As an alternative, person-centered approaches, that is, approaches focused on identifying subgroups of people based on their similarities on a set of variables (e.g., cluster analysis, latent profile analysis), allow to identify patterns of interrelations among variables for different, and meaningful, subgroups of individuals. In older adult populations, researchers have distinguished profiles of older adults based on diverse characteristics, such as diet patterns (28); food-related life satisfaction and health safety (14); loneliness and psychosocial risk (29); and active lifestyles (6). Most of this research, however, has been conducted before the pandemic. Further exploration is needed to understand the interrelations between protective and risk factors of life satisfaction in diverse older adults during the pandemic [e.g., (30, 31)].

### 1.1. Life and food-related life satisfaction and associated variables in older populations

A starting point to understand the heterogeneity of life satisfaction among older adults is the bottom-up theoretical approach to life satisfaction and the spillover hypothesis. According to the bottom-up approach, overall life satisfaction depends on the person's assessment of their different life domains (32). To complement this approach, the spillover hypothesis states that there is a positive correlation between life and domain satisfaction (33). Furthermore, according to Sirgy (34), one individual's experiences in one domain may not only spill over to their overall life satisfaction vertically (bottom-up or vertical spillover), but also to adjacent life domains, such as family, health, finances, and leisure (horizontal spillover). Therefore, as evidence shows, life domains are not only related to overall life satisfaction but also among one another [e.g., (35, 36)]. These dynamics require a research approach that can account for this complexity.

The literature on older adults' wellbeing reflects both the bottom-up approach and the spillover hypothesis, indicating the influence of several factors. These factors relate to living situation (25, 37–39); satisfaction with financial situation and income (25, 27, 37, 40–42); physical and mental health (3, 7, 25, 37); daily activities and leisure (6, 27, 37, 41); social support (27–29, 38, 39, 43); family interaction (3, 7, 26, 41); age (25, 27, 44); marital status (4, 25, 42, 44); friends and neighbors (3); and diet quality (6, 7, 11, 28, 37, 45). Most research in this regard, however, has been conducted in developed countries, in older adults with existing co-morbidities (37), and before the COVID-19 pandemic. Less is known about the conditions of older populations in developing regions, such as South American countries, and accounting for the context of the pandemic.

There is also evidence of horizontal spillover for food-related satisfaction. This construct has been associated with family (7, 12, 13, 15), social support networks (19), economic situation (14–16), physical and mental health (7, 14, 15), and perceived resources (15, 16). Sociodemographic characteristics have also been linked to food-related life satisfaction, such as sex (12, 14, 16, 19), age (12, 19), education (14), marital status, and area of residence (12).

The relation between life satisfaction and food-related life satisfaction is also heterogeneous. Studies on university students and adults in Chile show that subgroups from these populations have diverging levels of both types of satisfaction, and these subgroups also differ in eating habits, health, and sociodemographic characteristics. In these studies, groups with healthier eating habits had higher levels of life and food-related satisfaction (46, 47). These studies were replicated by Schnettler et al. (7) in a sample of Ecuadorian older adults. Their findings mirrored the above patterns, but notably, one profile showed high food-related life satisfaction with slightly poorer dietary quality, and these associations were mediated by a higher frequency of having lunch in the company of others. These discrepancies underscore the need to better understand how diverse living conditions contribute to older adults' subjective wellbeing.

Research conducted during the pandemic indicates relevant contributors and protective factors of older adults' life satisfaction. During the first year of the COVID-19 pandemic, Chen and Olsen (48) found that health, personal relationships, and standard of living were the most important domains of life that explained life satisfaction in older adults in Australia. Kivi et al. (23) reported that health and financial situation were important domains of life that predicted Swedish older adults' wellbeing in the early stage of the pandemic. In Spain, Díaz-García et al. (49) found that social relations remain one of the most essential aspects for wellbeing in older adults, above other aspects such as health and wealth. In Turkey, Onal et al. (43) found that perceived social support, high income, higher education degree, and residing in the Mediterranean region were factors associated with the increase in life satisfaction. In Belgium, Van Loon and Decancq (50) found that health, income, and social relations had the highest effect on older adults' wellbeing during the second year of the pandemic. These studies have explored the association between these variables, but there remains the need to assess the heterogeneity of these patterns of association in older adults, considering life and food-related life satisfaction, and related factors such as diet quality, social and financial resources, and sociodemographic characteristics. Identifying factors associated with membership to these profiles can inform tailored interventions (29), which may improve subsequent psychological wellbeing and decrease subsequent psychological distress in older adults (41).

### 1.2. This study

This study provides new insights about the heterogeneity of life and food-related life satisfaction in older adults in Latin America. As in other countries, the lifespan in Chile has extended and it is expected to increase from 77 to 80.7 years for the period 2020–2025 (22). According to estimations (22), 18% of the Chilean population are older adults, and it is expected that by 2050, older adults will make up 32% of the total population. By 2020, older adults in Chile reported an average of 9 years of schooling, and 82% did not have functional dependence on others. However, in normal conditions, there are individuals within this group at higher risk, such as those who live in long-term stay facilities for older adults (Establecimientos de Larga Estadía para Adultor Mayores, ELEAM). ELEAMs are residences or support centers for older adults who require a protected environment and differential care to maintain their health and functionality (51). The Chilean State also offers support to older adults via the Centers for Older Adults (Clubes de Adulto Mayor, CAM). These are daytime facilities for older adults who otherwise live independently, which provide support services, sociocultural activities, and active aging. CAMs seek to favor autonomy and maintain older adults in their families and communities (52).

During the COVID-19 pandemic, older adults in Chile represented 14.4% of total contagions of COVID-19, and they made up 50% of hospitalizations and 90% of deaths (53). Hence, special measures were implemented to protect this population. Diverse governmental and non-governmental organizations established a task force to address preventive and control measures for COVID-19 in ELEAMs and for those who remained on lockdown in their homes. Other measures to address the COVID-19 risk in this population were increasing the provision of physical and psychological health services and the implementation of economic measures (22). In the present study, the heterogeneity in older adults living in ELEAM, those who participate in CAM, and those who do not live in ELEAM or participate in CAMs was assessed.

Against this background, the present study adopted a person-centered approach to characterize older adults based on their satisfaction with life and food-related life, diet quality, financial wellbeing, and sociodemographic characteristics. The aims of this study were as follows: (a) to identify older adults' profiles based on their levels of satisfaction with life and food-related life; (b) to compare these profiles by characterizing older adults' diet quality, social support, financial wellbeing, and sociodemographic characteristics; and (c) to identify variables associated with belonging to the profile with higher levels of satisfaction with life and food-related life.

## 2. Method

### 2.1. Sample and procedure

The study design was descriptive and cross-sectional, and it was conducted in the Maule Region, Chile. This study considered the four main cities of this region: Linares, Curicó, Cauquenes, and Talca. A minimum sample size of 1,000 participants is recommended in studies using latent profile analysis (54). The final sample included 1,371 institutionalized and non-institutionalized individuals over the age of 60.

This study considered three independent samples. The first sample included 299 older adults between 60 and 105 years old (M = 80.6, SD = 9.5) who were institutionalized residents in ELEAMs. These participants were contacted by phone through the person in charge of each ELEAM by trained recruiters to answer the questionnaire in the ELEAM via face-to-face contact. The second sample was composed of 537 older adults between 60 and 93 years old (M = 73.8, SD = 6.2) who were non-institutionalized but had signed up for a CAM. For this sample, a two-stage sampling, stratified by clusters with incidental (casual) subsamples and (snowball) networks within the cluster, was applied. Each CAM was selected randomly within each stratum. CAMs listed in the Chilean National Register of Social Organizations for seniors as part of the National Service for Senior Citizens (SENAMA) were used are clusters. These older adults were contacted by phone through the director of each CAM by trained recruiters to answer the questionnaire online, in the CAM or their homes. Finally, the third sample was composed of 535 older adults between 60 and 98 years old (M = 71.6, SD = 8.2) who were neither institutionalized, residents at an ELEAM nor had signed up at a CAM (no ELEAM/no CAM). This third sample was obtained through convenience sampling. Data were collected through interviews conducted online or face to face in public spaces near parks, convenience stores, and supermarkets, or in the participants' residences, in the four main cities of the Maule region. There were no missing data. Trained interviewers administered the questionnaire either face to face or online, and they recorded the responses on the paper questionnaires or the online response platform, to ensure that all questions were answered.

For the three samples, the inclusion criteria were that individuals had no cognitive impairment according to the Mini-Mental State Examination (MMSE) test (55). Individuals were sampled according to quotas for age, sex, and socioeconomic level. Only seniors who voluntarily agreed to participate were surveyed, and the anonymity of the respondents was protected. All participants gave their informed consent before they participated in the study. The study was conducted in accordance with the Declaration of Helsinki, and the protocol was approved by the Ethics Committee of Universidad de Talca (Act N° 25/2021). The survey was conducted between January and August 2021.

### 2.2. Measures

#### 2.2.1. Satisfaction with Life Scale (SWLS)

The SWLS (10) is a five-item scale that comprises a single dimension to evaluate the person's assessment of their own life (example item: “*In most ways my life is close to my ideal”*). Respondents indicate their degree of agreement with each statement using a seven-point Likert scale ranging from 1 = completely disagree to 7 = completely agree. SWLS scores are obtained by summing up the scores from the five items. The Spanish version of the SWLS was used (56), which has shown good internal consistency in studies with Chilean older adults (18, 57). In this study, the SWLS showed good internal reliability (Cronbach's alpha = 0.84).

#### 2.2.2. Satisfaction with Food-related Life (SWFoL)

The SWFoL (11) is a unidimensional five-item scale that evaluates the person's overall assessment of their food and eating habits (example item: “*Food and meals are positive elements”*). Respondents indicate their degree of agreement with each statement using a six-point Likert scale, from 1 = completely disagree to 6 = completely agree. SWFoL scores are obtained by summing up the scores from the five items. The Spanish version of the SWFoL was used (56), which has shown good internal consistency in studies with Chilean older adults (15, 16, 18, 57, 58). In this study, the SWFoL showed good internal reliability (Cronbach's alpha = 0.90).

#### 2.2.3. Multidimensional Scale of Perceived Social Support (MSPSS)

The MSPSS (59) is composed of 12 items and three four-item subscales: FAMILY (e.g., “*My family really tries to help me”*), Friends (e.g., “*I can count on my friends when things go wrong”*), and others (e.g., “*There is a special person who is around when I am in need”*). Respondents rate each statement using a five-point Likert scale from 1 = completely disagree to 5 = completely agree. Scores for each subscale are obtained by summing up the scores from their four items. The validated Spanish version of the scale was used (60), which has shown good internal consistency in studies with Chilean older adults (60, 61). In this study, the three subscales showed good internal reliability (Cronbach's alpha Family = 0.88, Friends = 0.93, Others = 0.86).

#### 2.2.4. Food Quality Survey for the Elderly (FQSE)

The FQSE (62) is composed of 21 questions distributed in two subscales. The first subscale, Healthy eating habits, has 13 questions to explore the frequency of healthy habits and consumption of recommended food groups. Each question is scored on a Likert-type scale from Does not consume (1 point) to Suggested servings per day/week (5 points). The score of this subscale varies from 13 to 65 points (higher values indicate healthier eating habits). The second subscale, Unhealthy eating habits, is made up of eight questions, with a minimum score of 1 and a maximum of 5 per question (Likert-type scale) for the first seven questions, and a minimum score of 1 and a maximum of 3 for the last one. These questions address the quantity and frequency of consumption of foods or food groups identified as promoters of non-communicable chronic diseases. Each question is scored from bad eating habits (1 point) to not consuming (3 or 5 points as appropriate), with the total score of this subscale ranging from 8 to 38 points (higher values indicate healthier eating habits). The validated Spanish version of the scale was used, which has shown good internal consistency in studies with Chilean older adults (62). In this study, both subscales showed acceptable internal reliability (Cronbach's alpha Healthy eating habits = 0.70, Unhealthy eating habits = 0.69).

#### 2.2.5. Financial distress/financial wellbeing scale (FDFW)

The FDFW (63) is an eight-item scale that measures subjective financial distress/financial wellbeing. This scale uses the mean score of a 10-point Likert scale (ranging from 1 to 10), and it provides a score by combining responses to eight individual indicators (e.g., “*What do you feel is the level of your financial stress today?”*). Joo and Grable (64) state that this mean score is a valid and reliable measure of the latent construct of perceived financial distress/wellbeing. Higher mean scores entail higher levels of financial wellbeing. A Spanish version of this scale was used in this study, which showed good levels of internal consistency (α = 0.92) in studies in Ecuador (65). In this study, the FDFW scale showed good internal reliability (Cronbach's alpha = 0.92).

Participants were also asked about their age, sex, marital status, educational level, monthly income, and whether they had an ever-confirmed COVID-19 infection prior to the survey.

### 2.3. Data analysis

The current study used a three-step process to identify older adult profiles based on their levels of life and food-related life satisfaction. The first step was to group older adults based on their SWLS and SWFoL scores. A latent profile analysis (LPA) for continuous variables was used to estimate the number of profiles associated with SWLS and SWFoL scores of older adults (66). Although a previous study distinguished three typologies of older adults based on their SWLS and SWFoL scores in Ecuador (7), these authors used hierarchical cluster analysis to distinguish the types. This method poses the limitation that the choice of the number of clusters is arbitrary, while LPA allows for the statistical comparison of models to determine the number of clusters (67).

The LPA models were tested iteratively; that is, multiple latent profile models (1–5 groups) were estimated to find the best-fitting model using participants' SWLS and SWFoL scores and age, marital status, and education as covariates. The best-fitting model (i.e., the optimal number of profiles) was selected based on the Bayesian information criterion (BIC), the consistent Akaike's information criterion (CAIC), and the entropy test. Lower BIC and AIC scores indicate a better fit. Entropy refers to the degree of certainty regarding the inclusion of participants in one profile; entropy values above 0.80 are considered acceptable, as values near 1 mean a higher degree of certainty (66, 68). The sample size of the smallest profile was also evaluated as a small sample-size class (i.e., <1% and/or n <25) may have less precision and low power (69). The LPA was conducted using the Latent Gold 5.1 statistics software (Statistical Innovations Inc.).

After older adults were grouped into the three profiles based on their SWLS and SWFoL scores, the second step of the analysis involved characterizing these profiles based on statistical differences found in the variables concurrent with these scores. Pearson's chi-square test was applied to the discrete variables. A one-factor analysis of variance was used to examine whether profiles differed in terms of the older adults' life satisfaction, satisfaction with food-related life, age; perceived social support from family, friends, and others, financial wellbeing, and healthy and unhealthy eating habits and total score according to the FQSE. Continuous variables for which Levene's statistic indicated homogeneous variances (*p* < 0.05 or *p* ≤ 0.001) were subjected to Tukey's multiple comparisons test, while those with non-homogeneous variances (*p* < 0.05 or *p* < 0.001) were subjected to Dunnett's T3 multiple comparisons test.

In the third step of this analysis, characteristics that significantly differed across profiles were evaluated in a multivariate ordinal logistic regression model, to identify characteristics associated with belonging to the profile with higher levels of satisfaction with life and food-related life. Therefore, the profiles were introduced as the dependent variable in the model with the profile with higher levels of satisfaction with life and food-related life as the reference group. The−2 log of likelihood, Pearson and Deviance chi-squared, and Nagelkerke pseudo R squared were used to assess the goodness of fit of the multivariate ordinal logit model.

Pearson's chi-square test, one-factor analysis of variance, and the multivariate ordinal logistic regression model were conducted using the Statistical Package for Social Sciences (IBM SPSS) v. 23.

## 3. Results

### 3.1. Sample description

Table 1 shows the sociodemographic characteristics of the sample. The mean age of the participants was 74.4 years (range 60–105), and 58.4% were female. Most of the older adults were not institutionalized (78.2%) and were married, widowed, or single. Most of the participants had elementary and high school education and reported a monthly income lower than USD 562 (88.1%). A high proportion of participants lived in Talca, and most of them did not have an ever-confirmed COVID-19 infection prior to the survey.

**Table 1:** Sample characteristics (*n* = 1,371).

**Characteristic**		**ELEAM (*n* = 299)**	**CAM (*n* = 537)**	**No ELEAM/no CAM (*n* = 535)**	**Total sample (*n* = 1,371)**	***P*-value**
Age [Mean (*SD*)]^a^		80.6 (9.5)	73.8 (6.2)	71.6 (8.2)	74.4 (7.4)	<0.001
Sex (%)^b^	Male	39.5	39.1	45.2	41.6	0.089
	Female	60.5	60.9	54.8	58.4	
Monthly income (USD, %)^b^	<324	66.9	64.8	70.3	67.4	0.238
	324–561	21.7	20.7	20.2	20.7	
	562–898	6.4	7.6	6.4	6.9	
	899–1,359	3.3	3.5	1.5	2.7	
	More than 1,360	1.7	3.4	1.5	2.2	
Marital status (%)^b^	Married	6.7	51.2	50.8	41.4	<0.001
	Cohabiting	1.3	1.7	3.9	2.4	
	Separated	11.3	4.3	4.8	5.4	
	Divorced	5.0	5.8	7.5	7.3	
	Widowed	36.1	25.9	19.6	25.7	
	Single	39.5	11.2	12.3	17.8	
Education (%)^b^	Without formal schooling	16.4	0.7	5.6	6.1	<0.001
	Elementary	46.8	36.3	40.4	40.2	
	High school	24.4	39.4	38.0	35.5	
	University	12.4	23.6	16.1	18.2	
City of residence (%)^2^	Cauquenes	5.4	8.2	8.2	7.6	0.822
	Curicó	29.1	27.4	27.5	27.8	
	Linares	28.1	27.2	26.7	27.2	
	Talca	37.5	37.2	37.6	37.4	
Sick with COVID-19 (%)^b^	Yes	25.1	16.6	19.6	19.6	0.012
	No	74.9	83.4	80.4	80.4	

^a^*P*-value corresponds to the analysis of variance.

^b^*P*-value corresponds to the (bilateral) asymptotic significance obtained in Pearson's chi-square test.

Significant differences were found according to the living situation in terms of age, marital status, education (*p* < 0.001), and if older adults had an ever-confirmed COVID-19 infection prior to the survey (*p* < 0.005). There were no statistical differences in terms of sex (*p* >0.05), monthly income, and city of residence (*p* > 0.1). Older adults living in an ELEAM had an average age significantly higher than those who participated in a CAM and higher than those who did not live in an ELEAM nor participated in a CAM. Older adults who participated in a CAM had an average age significantly higher than those who did not live in an ELEAM nor participated in a CAM. Regarding marital status, older adults living in an ELEAM had a greater proportion of separated, widowed, and single participants, and a lower proportion of married participants. Older adults who participated in a CAM had a greater proportion of married and a lower presence of single participants, while older adults who did not live in an ELEAM nor participated in a CAM had a greater percentage of married and cohabiting and a lower proportion of single participants. Regarding educational level, older adults living in an ELEAM had a greater proportion of participants without formal schooling and a lower presence of participants with university studies. By contrast, older adults who participated in a CAM had a greater proportion of participants with university studies and a lower presence of participants without education. Finally, older adults living in an ELEAM had a greater proportion of participants who had an ever-confirmed COVID-19 infection, while the opposite trend was observed in older adults who participated in a CAM.

Table 2 shows the average scores and the correlations for satisfaction with life scale (SWLS), satisfaction with food-related life scale (SWFoL), perceived social support from family (PSSFa), friends (PSSFr) and others (PSSO), healthy and unhealthy eating habits according to the Food Quality Survey for Elderly (FQSE), and the total score of the FQSE along with the financial distress/financial wellbeing (FDFW) score. The SWLS average score indicated that participants were satisfied with their life, while the SWFoL average score showed that participants were extremely satisfied with their food-related life. The healthy eating habits average score indicated a poor diet quality, while the unhealthy eating habits average score was slightly above the cutoff score indicating good diet quality. The total average score for the FQSE indicates poor diet quality, although it was close to the cutoff score indicating good diet quality (62). The average score for FDFW indicated moderate financial distress/moderate financial wellbeing (63).

**Table 2:** Descriptive statistics and correlations for older adults' satisfaction with life (SWLS), satisfaction with food-related life (SWFoL), perceived social support from family (PSSFa), perceived social support from friends (PSSFr), perceived social support from others (PSSO), healthy and unhealthy eating habits according to the Food Quality survey for Elderly (FQSE), and financial distress/financial wellbeing (FDFW) (*n* = 1,371).

	**M (SD)**	**Correlations**								
		**1**	**2**	**3**	**4**	**5**	**6**	**7**	**8**	**9**
1. SWLS	26.9 (6.9)	-	0.412^***^	0.375^***^	0.279^***^	0.383^***^	0.064^*^	−0.026	0.042	0.392^***^
2. SWFoL	26.3 (4.8)		1	0.268^***^	0.212^***^	0.312^***^	0.091^**^	−0.004	0.076^**^	0.235^***^
3. PSSFa	22.4 (6.5)			1	0.428^***^	0.711^***^	−0.004	−0.072^**^	−0.037	0.240^***^
4. PSSFr	19.5 (7.6)				1	0.488^***^	0.018	−0.079^**^	−0.022	0.209^***^
5. PSSO	23.3 (5.7)					1	0.018	−0.057^*^	−0.012	0.263^***^
6. Healthy eating habits	46.2 (5.6)						1	0.053^*^	0.879^***^	0.140^***^
7. Unhealthy eating habits	32.7 (3.1)							1	0.523^***^	−0.053^*^
8. FQSE (total)	78.9 (6.5)								1	0.094^***^
9. FDFW	6.1 (2.3)									1

Data analysis was performed using Pearson's correlation coefficient. ^*^p < 0.05, ^**^p < 0.01, ^***^p < 0.001.

Most of the correlations were significant and in the expected direction, except the correlation between SWLS and unhealthy eating habits scores, and between the SWFoL and unhealthy eating scores. Similar results were found for the correlations between perceived social support from family, friends, and others and the healthy eating habits scores and the FQSE total score. By contrast, the average scores of three types of social support negatively correlated with the unhealthy eating habits score. This means that social support from family, friends, and others might not help older adults to have healthy eating habits, but they might help them to avoid unhealthy eating habits.

### 3.2. Latent profile analysis of older adults

An initial run of one to five clusters was analyzed with the SWLS and SWFoL scores. The three-cluster model (Table 3) had the best fit because it had the lowest BIC and CAIC values and a value in the entropy test above 0.80 (66, 68). The sample size of the smallest profile was >5%; therefore, it had adequate precision and power (69). The Wald statistics associated significance levels were below 0.001, which is evidence that the SWLS (robust Wald statistic = 68.01, *p* = 1.7e-15) and SWFoL (robust Wald statistic = 83.27, *p* = 8.3e-19) scores were useful for segmenting older adults. The percentage of variance explained was 50.93% for SWLS and 30.98% for SWFoL.

**Table 3:** Summary of latent profile cluster models.

	**LL**	**BIC (LL)**	**CAIC (LL)**	**Npar**	**Entropy**	**Classification error**
1-Cluster	−7,352.96	15,103.20	15,158.21	55	1.00	0.00
2-Cluster	−7,221.85	14,891.50	14,953.50	62	0.92	0.13
3-Cluster	−7,170.60	14,839.61	14,908.61	69	0.97	0.14
4-Cluster	−7,155.15	14,859.27	14,935.27	76	0.74	0.18
5-Cluster	−7,143.65	14,886.83	14,969.82	83	0.73	0.22

LL, Log-likelihood; BIC (LL), Bayesian information criterion based on the log-likelihood; CAIC (LL), Consistent Akaike's Information Criterion; Npar, Number of parameters.

**Figure 1:**
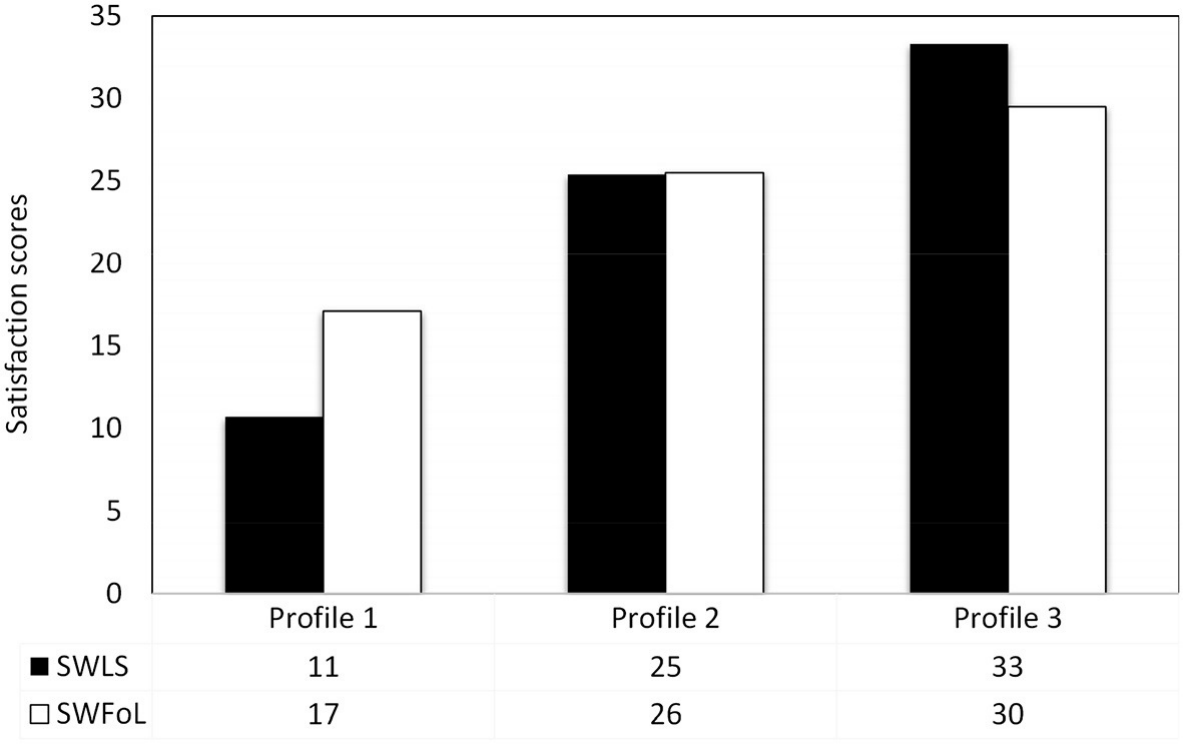
Profiles based on life satisfaction and satisfaction with food-related life in older adults. Profile 1: “Unsatisfied with their life, somewhat satisfied with their food-related life”. Profile 2: “Somewhat satisfied with their life, satisfied with their food-related life”. Profile 3: “Extremely satisfied with their life and food-related life”. Scores for the Satisfaction with Life Scale and the Satisfaction with food-related scale were subjected to Dunnett's T3 multiple comparisons test.

The resulting profiles are described below, labeled based on their average SWLS and SWFoL scores (Figure 1). Table 4 shows the scores of the continuous variables with statistical differences used to describe older adult profiles, while Table 5 shows differences between profiles according to the discrete variables.

**Table 4:** Differences between the three profiles according to age, financial distress/financial wellbeing, and perceived social support from family, friends, and others in older adults.

	**Profile 1**	**Profile 2**	**Profile 3**	**F**	***P*-value**
Profile size (%)	5.40	65.06	29.54		
Age^a^	71.05 b	74.96 a	73.84 ab	8.575	<0.001^***^
Financial distress/financial wellbeing^b^	4.09 c	5.80 b	7.05 a	77.984	<0.001^***^
Perceive social support from					
Family^b^	15.81 c	21.82 b	24.78 a	27.802	<0.001^***^
Friends^a^	12.32 c	19.12 b	21.55 a	21.212	<0.001^***^
Significant Other^b^	16.62 c	22.77 b	25.70 a	18.995	<0.001^***^

^a^Different letters in the line indicate significant differences according to Tukey's multiple comparisons test. “a” is significantly higher than “b.” “b” is significantly higher than “c.” ^***^p < 0.001.

^b^Different letters in the line indicate significant differences according to Dunnett's T3 multiple comparisons test. “a” is significantly higher than “b.” “b” is significantly higher than “c.” ^***^p < 0.001.

Profile 1: “Unsatisfied with their life, somewhat satisfied with their food-related life”.

Profile 2: “Somewhat satisfied with their life, satisfied with their food-related life”.

Profile 3: “Extremely satisfied with their life and food-related life”.

**Table 5:** Percentage of older adults in the three profiles by socioeconomic differences, that is, living situation, marital status, COVID-19 infection, and city of residence.

	**Profile 1**	**Profile 2**	**Profile 3**
*Living situation*		*p*<*0.001*	
ELEAM	51.4	24.1	11.4
CAM	4.1	45.3	32.1
No ELEAM, no CAM	44.6	30.6	56.5
*Marital status*	*p*<*0.001*
Married	14.9	40.8	47.4
Cohabiting	5.4	1.0	5.9
Separate	10.8	5.8	3.5
Divorced	12.2	7.0	6.9
Widowed	27.0	26.1	24.4
Single	29.7	19.2	12.6
*COVID-19 infection*	*p = 0.010*
Yes	31.3	20.2	16.3
No	68.9	79.8	83.7
*City of residence*		*p = 0.001*	
Cauquenes	6.8	7.7	7.4
Linares	36.5	24.7	31.1
Curicó	17.6	26.7	32.1
Talca	39.2	40.9	29.4

*P*-value corresponds to the (bilateral) asymptotic significance obtained in Pearson's chi-squared test.

Profile 1: “Unsatisfied with their life, somewhat satisfied with their food-related life”.

Profile 2: “Somewhat satisfied with their life, satisfied with their food-related life”.

Profile 3: “Extremely satisfied with their life and food-related life”.

#### 3.2.1. Latent profile 1

*Older adults unsatisfied with their life, somewhat satisfied with their food-related life* (5.40% of the sample). This profile had the lowest average scores for SWLS (*F* = 956.691 *p* < 0.001), SWFoL (*F* = 370.505 *p* < 0.001) (Figure 1), financial wellbeing, and perceived social support from family, friends, and others (*p* < 0.001, Table 4). Profile 1 had a significantly higher proportion of institutionalized older adults living in an ELEAM (*p* < 0.001), single (*p* < 0.001), and who had an ever-confirmed COVID-19 infection prior to the survey (*p* < 0.05).

#### 3.2.2. Latent profile 2

*Older adults somewhat satisfied with their life, satisfied with their food-related life* (65.06% of the sample). The SWLS and SWFoL average scores in Profile 2 were significantly lower than Profile 3 but significantly higher than Profile 1 (Figure 1). Older adults from this profile had the highest average age, although they did not differ from older adults in Profile 3 (*p* < 0.001). The average scores for financial wellbeing and perceived social support from family, friends, and others were significantly lower than those of Profile 3, but significantly higher than Profile 1 (Table 4). Profile 2 had a significantly higher presence of institutionalized older adults (living in an ELEAM) and not institutionalized that participated in a CAM. This profile had a lower percentage of older adults living in Linares (*p* < 0.01) and a higher proportion living in Talca (Table 5).

#### 3.2.3. Latent profile 3

*Older adults extremely satisfied with their life and food-related life* (29.54% of the sample). This profile had SWLS and SWFoL average scores significantly higher than Profiles 1 and 2 (Figure 1). Average scores for financial wellbeing and perceived social support from family, friends, and others were significantly higher than those of Profiles 1 and 2 (Table 4). Profile 3 had a significantly higher presence of non-institutionalized older adults who did not participate in a CAM. This profile had a higher proportion of married and cohabiting older adults. Profile 3 had a lower percentage of older adults living in Talca and a higher proportion living in Linares (Table 5).

No significant differences between the profiles were found in sex (*p* = 0.116), education (*p* = 0.797), monthly income (*p* = 0.725), nor in the healthy eating habits (*p* = 0.816), unhealthy eating habits (*p* = 0.691) and in the FQSE total scores (*p* = 0.981).

We also explored differences in the responses to the 21 items of the FQSE. Results showed that Profile 3 had a greater proportion of older adults who had breakfast every day, while Profile 1 had a higher presence of older adults who had breakfast less than once a week (Chi^2^ = 16.231, *p* = 0.039). Profile 3 had a higher proportion of older adults who consume two servings a day of fresh fruits (Chi^2^ = 26.993, *p* = 0.001). Regarding fish consumption (fresh/frozen/preserved, but not fried), Profile 3 had a greater presence of older adults who consumed one serving a week, while Profile 1 had a greater proportion of older adults who did not consume fish (Chi^2^ = 22.333, *p* = 0.004). Profile 3 also had a higher presence of older adults who consumed one serving a week of legumes and a lower presence of older adults who consumed two servings a week (Chi^2^ = 16.897, *p* = 0.031). Regarding the consumption of oatmeal or whole wheat bread, Profile 1 had a higher presence of older adults who consumed one serving a day and a lower proportion who did not consume, Profile 2 had a higher presence of older adults who consumed three servings a day, while Profile 3 had a greater proportion of older adults who did not consume this type of food (Chi^2^ = 34.138, *p* < 0.001). While Profile 3 had a higher proportion of older adults who never consumed a meal plus fruit or salad at dinnertime, Profiles 1 and 2 had a greater presence of older adults who consumed these foods daily (Chi^2^ = 38.009, *p* < 0.001). Profiles 1 and 3 had higher proportions of older adults that consumed four glasses or more a day of water or liquids (herbal waters, fruit juices, tea, mate, Chi^2^ = 20.831, *p* = 0.008). Profile 3 had a higher proportion of older adults who had three meals a day, while Profile 1 had a higher presence of older adults who had four meals and one snack per day. Finally, Profile 2 had a higher proportion of older adults who did not add salt to foods before tasting them, while in Profile 1, there was a higher presence of older adults who always added salt to foods before tasting them (Chi^2^ = 11.260, *p* = 0.024).

### 3.3. Variables associated with higher satisfaction with life and food-related life

The results of the multivariate ordinal logit model to identify characteristics associated with belonging to the profile with higher levels of satisfaction with life and food-related life (Profile 3) are shown in Table 6. Although the analysis plan stated that only characteristics that significantly differed across profiles would be incorporated in the model, we found statistical differences between the profiles in some eating habits, and thus, we also incorporated the average total score of the FQSE in the model.

**Table 6:** Results of the ordinal logit regression model generated to measure satisfaction with life and satisfaction with food-related life of older adults.^a^

	**Estimation**	**Wald**	**Lower limit**	**Upper limit**
Profile 1^b^	3.932^***^	15.936	2.001	5.862
Profile 2^b^	8.697^***^	72.566	6.696	10.698
**Explanatory variables**				
Food Quality Survey for Elderly (FQSE)	0.025^*^	5.794	0.005	0.045
Financial distress/financial wellbeing (FDFW)	0.278^***^	87.631	0.220	0.336
Age	0.019^*^	5.509	0.003	0.034
Perceived social support from friends (PSSFr)	0.019^*^	3.976	0.000	0.037
Perceived social support from significant other (PSSO)	0.088^***^	27.127	0.055	0.122
ELEAM	−1.711^***^	66.474	−2.122	−1.300
CAM	−0.654^***^	22.709	−0.923	−0.385
No Eleam, no CAM	0^c^			
Cauquenes	0.451	3.449	−0.025	0.927
Linares	0.611^***^	15.453	0.307	0.916
Curicó	0.485^**^	10.097	0.186	0.783
Talca	0^c^			
COVID-19 infection = No	−0.348^*^	4.937	−0.656	−0.041
COVID-19 infection = Yes	0^c^			
Nagelkerke adjusted R^2d^		0.305		
−2 Logarithm of the likelihood (-2LL)^e^		1,805.298^***^		
Pearson chi-squared (df)		2,442.372 (2,724)		
Deviance chi-squared (df)		1,805.298 (2,724)		

^a^Significant variables at ^*^p < 0.05, ^**^p < 0.01, ^**^p < 0.001 based on the Wald statistic.

^b^Value of the threshold or limit parameter (cut parameter). There are two threshold parameters because there are three categories for the dependent variable Profiles.

^c^This parameter is at zero because it is redundant. This is a comparison category for each explanatory variable in the model.

^d^Nagelkerke's R^2^ is a statistical proxy of the determination coefficient (Pseudo-R^2^) in the logit model.

^e^Significant model at the level ^***^p < 0.001 for the −2 log-likelihood.

The−2 log-likelihood and the Pearson and Deviance chi-squared indicated a good fit to the data, while the Nagelkerke pseudo-R^2^ value was >0.3. According to these results, older participants (β =0.019), those who had a higher average score on the FSQE (β = 0.025) and those who experienced greater financial wellbeing (β = 0.278) were more likely to belong to Profile 3 (*older adults extremely satisfied with their life and food-related life*). Similarly, participants who reported higher perceived social support from friends (β = 0.019) and significant others (β = 0.088) were more likely to belong to Profile 3. The same effect was obtained if older adults living in Linares (β = 0.611) and Curicó (β = 0.711) were compared with those living in Talca. By contrast, the likelihood that participants belonged to Profile 3 decreased if they were institutionalized older adults living in an ELEAM (β = −1.711) and non-institutionalized older adults participating in a CAM (β = −0.654) compared with non-institutionalized older adults who did not participate in a CAM. Similarly, the likelihood that older adults belonged to Profile 3 decreased if they had an ever-confirmed COVID-19 infection prior to the survey (β = −0.348) compared with those who did not.

A significant interaction was observed between the FQSE scores and living conditions. Healthier eating habits increased the likelihood of belonging to Profile 3. This likelihood decreased if the individual lived in an ELEAM and increased if they attended a CAM, compared with non-institutionalized older adults who did not participate in a CAM. An interaction was also found between financial wellbeing and living conditions. Higher financial wellbeing increased the likelihood of belonging to Profile 3, and it was less likely if the individual attended a CAM, compared with non-institutionalized older adults who did not participate in a CAM.

## 4. Discussion

The first aim of this study was to identify profiles of older adults based on their levels of life satisfaction and food-related life satisfaction. We used latent profile analysis (LPA) to distinguish three latent profiles based on participants' scores of life and food-related life satisfaction. Profile 1 comprised *older adults unsatisfied with their life, somewhat satisfied with their food-related life* (5.40% of the sample); Profile 2 included *older adults somewhat satisfied with their life, satisfied with their food-related life* (65.06%); and Profile 3 comprised *older adults extremely satisfied with their life and food-related life* (29.54%).

A closer examination of these profiles showed further differences, discussed in detail below in response to the other two aims of this study. Overall, older adults belonging to Profile 1 were younger, had the lowest average scores in social support from family, friends, and others and in financial wellbeing; a significant proportion of this group resided in an ELEAM, were single, and had an ever-confirmed COVID-19 infection. Profile 2 had medium average scores in social support and financial wellbeing, while a greater proportion of participants resided in an ELEAM, participated in a CAM, and resided in Talca. Finally, older adults from Profile 3 had the highest average scores in social support and financial wellbeing, while a greater proportion of participants did not reside in an ELEAM nor participated in a CAM, they were married or cohabitant, and resided in Curicó.

These findings provide evidence that older adults constitute a heterogeneous population (4–6, 14, 28, 29). We also show that the relation between life satisfaction and food-related life satisfaction is heterogeneous (7, 46, 47). These results are in line with research showing profiles with low, medium, and high levels of satisfaction with life and food-related life in samples of undergraduate students and adults in Chile (46, 47). However, our results display a different pattern of profiles in comparison with a previous study in a sample of Ecuadorian older adults, in which two groups had high levels of life and food-related life satisfaction and the third one had medium levels of both satisfactions (7). Given this comparison, we hypothesize that heterogeneity in the relationship between life satisfaction and food-related life satisfaction is associated with the sociocultural context rather than with age.

### 4.1. Psychosocial variables associated with life and food-related life satisfaction

The second aim of this study was to characterize the profiles of older adults according to their diet quality, social support, financial wellbeing, and sociodemographic characteristics. These findings illustrate vertical and horizontal spillover, that is, that participants' experiences in one domain spill over to their overall life satisfaction, and are simultaneously linked to adjacent life domains (32, 34) such as family, friends, finances, and living conditions. These differences are examined below.

Regarding living conditions, Profiles 1 and 2 were composed of a higher proportion of older adults living in an ELEAM, and who showed lower levels of life and food-related life satisfaction in comparison with Profile 3, which was composed of a greater proportion of older adults who lived independently. Similarly, Yan et al. (39) found that community-dwelling older adults have higher levels of life satisfaction and social support than nursing home residents. Indeed, social support has been consistently associated with higher levels of life satisfaction in pre-pandemic studies (27–29, 38, 39) and during the pandemic (43, 48–50). Moreover, our results further contribute to this distinction by showing that older adults who live independently (Profile 3) have also higher levels of food-related life satisfaction than nursing home residents (Profiles 1 and 2). High levels of social support may also play a role in this case, as previous studies have positively linked social support and food-related life satisfaction in older adults in different countries (12, 13, 15, 19). Overall, the living conditions of older adults in our sample appear to be key, directly or indirectly (e.g., via social support), in maintaining their satisfaction with life and with food-related life.

Social support in this study was analyzed separately by family, friends, and others. Older adults from Profiles 1 and 2 reported lower perceived social support from these three sources than older adults from Profile 3. The lower perceived family support in Profile 1 may be associated with pandemic-specific measures. From the start of the pandemic in March 2020 to October 2020, the Chilean Ministry of Health prohibited visiting hours for older adults residing in an ELEAM. Since October 2020, family visits were authorized under strict sanitary measures, and these measures were relaxed in October 2021 (70). Hence, older adults in Profile 1 were the least likely from the three profiles to have frequent direct contact with their families during lockdowns.

Older adults from Profile 2 reported higher social support from friends and others than those from Profile 1 but also reported lower social support than those from Profile 3. Profile 2 had a higher proportion of older adults who did not live in an ELEAM but participated in a CAM, which offers sociocultural activities among other services (52) that could increase perceived social support from friends and others. This characteristic of Profile 2 contradicts studies showing that active participation in senior citizen centers is linked to higher levels of quality of life (6, 29). We hypothesize that this result is also associated with the pandemic. During 2020 and 2021, older adults may have retained their CAM membership, but their attendance and engagement were low due to the lockdown and the risk of COVID-19 infection (70). Taken together, these profile differences suggest that during the pandemic older adults living in ELEAM (Profiles 1 and 2) received less family support, while older adults not living in an ELEAM but involved in a CAM (Profile 2) received less support from friends and others. The loss of social support, and prolonged social isolation, may increase feelings of loneliness and decrease psychological wellbeing (4, 20), which may be reflected in their lower life and food-related life satisfaction.

By contrast, although the confinement measures were more restrictive for all older adults in Chile (70), older adults living independently may have had greater opportunities to maintain or even increase their social support from family, friends, and others during the pandemic. For instance, these older adults may have had their children and neighbors looking after them and running errands for them (e.g., leaving groceries at the door to be collected and disinfected by older adults) to keep them safe. This social support from friends and neighbors, in turn, may be associated with higher levels of life satisfaction (3). The association patterns of Profile 3 also suggest that this type of support is linked to higher levels of satisfaction with food-related life. The coexistence of these two factors may be explained by the social dimension of food purchasing, preparation, and consumption (11, 71).

Other differences in social support between profiles relate to marital status. Studies show that married or cohabiting older adults report higher levels of satisfaction with life (4, 25, 42) and with food-related life (12) than those who are single. Accordingly, in our study, Profile 1 (the least satisfied profile) had a higher proportion of single older adults, while Profile 3 (the most satisfied profile) had a greater proportion of married or cohabiting older adults. Moreover, for partnered individuals, good relations with the spouse (e.g., enjoying spending time together) also increase the individual's life satisfaction (72). On the contrary, Hansen et al. (4) reported that singlehood might have been riskier for older adults as they may have been particularly isolated during lockdown. Being married or cohabiting may have also enhanced some factors that contribute to food-related life satisfaction, such as eating in company (e.g., with the spouse) and a positive atmosphere during family meals (7, 12, 13, 15, 16, 36).

Contrary to the expectations, the profiles did not differ in their healthy eating habits, unhealthy eating habits, and total average score on the FQSE. Moreover, the differences found in some of the items of the FQSE showed that the three profiles had adequate and inadequate eating habits. These results contradict previous findings linking healthier diets to higher levels of life (6, 7, 11, 28, 37, 45) and food-related life satisfaction (2, 7, 13, 14, 17, 19) in older adults. However, satisfaction with food-related life not only encompasses nutritional issues but also the social dimension of food, pleasure, and positive emotions (7, 71). A study with Polish older adults showed that life and food-related life satisfaction are higher for those who focus on health, pleasure, and the social dimension of food rather than on exclusively health factors (17). Other studies with older adults show that food emotions and enjoyment are also relevant for their eating habits and quality of life (45, 73) and that older adults consider the hedonic aspect of eating very important (2). For instance, de Albuquerque et al. (12) found that higher levels of satisfaction with food-related life in Chilean older adults were associated with pleasant food activities such as drinking beer and eating at restaurants with family or friends. Hence, both nutritional and pleasure aspects of eating should be addressed when studding satisfaction with life and food-related life in older adults.

As our results did not show differences in diet quality among the distinguished profiles, we hypothesize that profile differences in life and food-related life satisfaction may be associated with the pleasure experienced by the older adults when eating. Evidence shows that negative emotions such as fear and anxiety emerged during the pandemic and increased people's risk perception, which in turn drove them to shift their attitudes toward a healthier diet as a way to boost their immune systems (74). Yet during this period pleasure may have been more salient than healthfulness for older adults when choosing foods. This tendency may be a reaction to the restriction of other pleasurable activities, such as spending time with friends and family (22, 23), or a coping strategy to deal with the increased feelings of loneliness (4, 20). Another possible explanation for the null differences in diet quality may be associated with the low monthly income of older adults in the sample. A systematic review by Caso and Vecchio (75) suggests that income and the price of healthy foods are among the main factors that influence older adults' healthy food choices. Given their reported income, older adults in our sample may be unable to afford healthier foods. However, given other results in this study, it appears that a healthy diet is not a significant contributor to satisfaction with life and food-related life in Chilean older adults.

Profiles also differed in terms of financial wellbeing. Following categories by Prawitz et al. (63), Profile 1 (the less satisfied profile) showed high financial distress/poor financial wellbeing; Profile 2 (medium levels of satisfaction) had moderate financial distress/moderate financial wellbeing; and Profile 3 (the most satisfied profile) had low financial distress/good financial wellbeing. These findings are consistent with studies conducted pre-pandemic (25, 27, 37, 40–42) and during the pandemic (23, 43, 50), showing a positive association between financial wellbeing or financial resources and life satisfaction in older adults. Nevertheless, these results may be paradoxical given that 67.4% of the total sample reported a monthly income of less than USD 324, while non-statistical differences in the monthly income between profiles were found. Our findings thus contradict studies associating greater financial wellbeing with higher income [e.g., (40, 65)].

Considering these results, we hypothesize two explanations. First, older adults received financial aid, food, and medicines from the State during 2020 and 2021 (22). These types of support may have alleviated the financial distress of older adults who lived independently, mainly found in Profiles 2 and 3. Second, financial wellbeing has also been associated with life satisfaction via social support (42, 65). This mechanism suggests that the more positively older adults perceived their financial situation, the closer their relationships are with family and friends, which in turn increases life satisfaction. Indeed, Profile 1 reported both the lowest financial wellbeing and the lowest perceived social support from family, friends, and others, Profile 2 had higher scores in these measures than Profile 1, and Profile 3 had higher scores than Profiles 1 and 2. Moreover, the profiles differed in marital status, as previously discussed. Being married or cohabiting (Profile 3) may entail a second income from the partner or spouse that increases the availability of financial resources for the household, thus increasing the individual's financial wellbeing. A better perceived economic situation was also linked to food-related life satisfaction in these profiles, in line with previous findings (14–16). However, our results suggest that this positive association does not relate to access to healthier diets (7, 14, 46) but to the hedonic and social dimension of food (2, 17, 45). Overall, these results show that financial wellbeing and distress are related to degrees of vulnerability in older adults. We found the highest financial distress in Profile 1 with the highest proportion of older adults living in an ELEAM, the most vulnerable group of older adults in Chile (51).

### 4.2. Sociodemographic variables associated with life and food-related life satisfaction

Our results only showed statistical differences between older adult profiles in terms of age. Studies have shown a positive association between age and life satisfaction [e.g., (44, 76)], and a negative association between food-related life satisfaction and age (12, 19). Findings from other studies, however, have questioned the traditional “U shape” of life satisfaction, that is, that it decreases in middle age and increases in older age (25, 27). In keeping with the latter evidence, we did not find a consistent pattern in the relation between these variables. These mixed results, however, may also be associated with the cross-sectional design of our study and others on life satisfaction. Hudomiet et al. (26) found that cross-sectional life satisfaction increases between age 65 and 71 and is flat thereafter, but, when measured longitudinally, life satisfaction significantly declines with age and the life satisfaction declining rate accelerates with age.

There was also a negative association between satisfaction with food-related life and age in the profiles, as reported in other studies (12, 19). This association may be explained by physiological barriers that some older adults face to have an adequate diet quality and enjoy eating, such as chronic illness, difficulties in swallowing, decline in taste and sense of smell, and lack of appetite (75). Our results may be reflecting this age-independent variation; that is, although older adults from Profile 1 had a lower average age than those in Profile 2 and they did not differ from Profile 3, a greater proportion of older adults from Profile 1 live in an ELEAM. Differences in satisfaction with food-related life between profiles may be thus associated with older adults' vulnerability rather than their chronological age.

Profiles also differed by COVID-19 infection. Profile 1 had a higher proportion of older adults with an ever-confirmed COVID-19 infection, compared with the other two profiles. This result is consistent with studies showing a higher rate of COVID-19 infection in older adults in long-term care facilities (77), such as ELEAM in Chile. The higher prevalence of COVID-19 in Profile 1 may also explain the lower levels of life and food-related life satisfaction in older adults from this profile. Older adults are more likely to experience a more severe COVID-19 infection and potentially greater concerns about its consequences due to their age and their likelihood of pre-existing health conditions (78). Evidence also shows a strong association between COVID-19 and subsequent psychological distress, depression, anxiety, and lower life satisfaction for people aged 50 years and older (78, 79). In older adults, lower levels of food-related life satisfaction have also been associated with a higher number of days with mental health problems (7, 15). Finally, COVID-19 has direct consequences for food-related life, such as loss or alteration of taste and smell and gastrointestinal side effects (80), which may be affecting older adults predominantly in Profile 1.

Regarding differences in the city of residence between profiles, previous studies during the pandemic have reported different levels of life satisfaction associated with city size, living in rural or urban areas, and region of residence. These factors are linked to specific conditions experienced during the health crisis, such as the proportion of inhabitants infected with COVID-19, population size, and degree of mobility (81). In this regard, although Linares reported a higher cumulative incidence rate of COVID-19 infection than Talca, this latter city had a larger population and had longer mandatory lockdowns than Linares during 2021 (53). Our findings provide support to studies reporting higher levels of life satisfaction in smaller cities and in those with fewer mobility restrictions during the pandemic (43, 81), namely, Profile 2 had a higher proportion of older adults living in Talca, and Profile 3 had a higher proportion of older adults living in Linares.

City of residence may also help explain the higher level of satisfaction with food-related life found in Profile 3 compared with Profile 2. Both profiles were composed of higher proportions of older adults living independently than Profile 1. For these older adults, longer lockdowns (i.e., in Talca) and time restrictions for grocery shopping and other errands may have hampered the purchase of their desired foods, which in turn may have negatively affected their satisfaction with food-related life. This impact may have occurred regardless of whether the food was purchased by the older adults themselves or by people from their support networks (e.g., children or neighbors).

### 4.3. Protective factors of life and food-related life satisfaction

The third aim of our study was to identify variables associated with belonging to the profile with higher levels of satisfaction with life and food-related life, that is, Profile 3. According to the results of the multivariate ordinal logit model, most of the variables identified are those that differed between profiles; these variables can be considered risk factors when they decrease the likelihood of belonging to Profile 3, and protective factors when they increase this likelihood.

In this regard, the likelihood of belonging to Profile 3 decreased if older adults resided in an ELEAM or participated in a CAM and if they had an ever-confirmed COVID-19 infection prior to the survey. By contrast, the likelihood of belonging to Profile 3 increased if older adults lived in smaller cities (Linares and Curicó, compared with those living in Talca, the capital city of the Maule Region), if they had higher financial wellbeing, higher social support from friends and others, and if they had a higher score in the FQSE. The likelihood of belonging to Profile 3 also increased with age, though this finding may be related to the cross-sectional design of this study, as previously discussed. Results of the ordinal logit model also allow us to identify the most influential variables in these relations. Among risk factors, living situation (i.e., living in an ELEAM or a CAM) had a higher influence than that of an ever-confirmed COVID-19 infection prior to the survey. Among the protective factors, the most influential variable was the city of residence, followed by financial wellbeing and perceived social support from others, while diet quality, age, and perceived social support from friends were less influential variables. Therefore, results from the multivariate ordinal logit model can guide health authorities and authorities in charge of older adults to prioritize the variables in which they can intervene to improve levels of life and food-related life satisfaction in this population.

Of note, family support and marital status did not influence the likelihood of belonging to Profile 3, although profiles differed in their perceived support from family and marital status. These findings contradict research that associated family relationships and support with higher levels of life (3, 7, 41) and food-related life satisfaction (7, 12, 13, 15, 19) in older adults, as well as the evidence showing that married or cohabiting older adults report higher levels of satisfaction with life (4, 25, 42) and with food-related life (12) than those who are single. Other studies, before (3) and during the pandemic (49), have also reported that family relations are the most important contributors to life satisfaction over relations with friends and others. However, family support and being married or cohabiting may not influence the likelihood of experiencing higher life and food-related life satisfaction because, as evidence suggests, these are previously established networks, compared with friendships that may require more effort to sustain (42). Older people in more vulnerable conditions (e.g., Profiles 1 and 2) may not be able to maintain friendships or relationships with others, such as neighbors, compared with those who live more independently (Profile 3). For these older adults, the ability to sustain social relations outside of the family may thus function as a positive influence (and a protective factor) for their life and food-related life satisfaction.

Finally, although profiles did not differ in their diet quality, the likelihood of belonging to Profile 3 increased if they had a higher average score on the FSQE. This finding was expected based on studies linking healthier eating habits with higher life and food-related life satisfaction (2, 6, 7, 11, 12, 14, 17, 19, 28, 37, 45). In the total sample, the unhealthy eating average score was slightly above the cut-off score indicating good diet quality, while the healthy eating habits average score indicated poor diet quality. This result may be associated with specific healthy eating habits of older adults from Profile 3, such as having breakfast daily and a higher frequency of consumption of fruits, which has been associated with higher life and food-related life satisfaction (7, 14, 46). Nevertheless, diet quality constitutes a generalized problem in the sample, which should be addressed mainly by fostering healthy eating habits that allow older adults to enjoy the social and hedonic dimension of food.

### 4.4. Limitations

We acknowledge the limitations of this study. The first one is that these results may not be generalizable to the population of older adults in Chile; the sample was non-probabilistic and pandemic measures and resources provided by the State and communities may have differed from one region to the other. In addition, the sample had a higher rate of contagions of COVID-19 (19.6%) than the total rate of Chile (14.4%) during the pandemic (53). Another limitation is that our questionnaire did not explore the social and hedonic dimensions of eating, such as with whom older adults share their mealtimes and its frequency, and the qualities of the food they consumed (taste, texture, healthfulness, among others). These dimensions appear to require as much attention as the nutritional aspects of food consumption. A third limitation is that the questionnaire did not explore older adults' personal and household income to better understand the objective conditions (and their consequences) related to financial wellbeing. The questionnaire also did not probe who were the “others” who provided support outside the circles of family and friends. In addition, self-rated mental and physical health has been related to life satisfaction and financial distress [e.g., (57, 65)], but these variables were not assessed in the questionnaire. Finally, these profiles may respond to the specific context of COVID-19, and profiles may vary in the aftermath of the pandemic.

### 4.5. Implications for research and policymaking

Despite its limitations, this study provides valuable knowledge on the heterogeneity among older adults' populations. This study included a large sample of older adults with different levels of vulnerability to represent the diverse conditions that this population has experienced during the second year of the COVID-19 pandemic in Chile. These findings have implications for research as they underscore the importance of advancing knowledge about older adult populations from a person-centered approach. In this sense, our results underscore the need for recognizing groups of older adults based on diverse characteristics and conditions outside of chronological age. Further questions arise from our findings regarding contextualized experiences in older age in terms of antecedents and correlates of social and pleasurable activities around food, financial security, living conditions (e.g., degree of autonomy, access to institutional resources), and perceived social support. Moreover, in Chile and other countries, research can explore the development of food and diets that are both nutritious and palatable within culturally valued foods.

As stated by the World Health Organization (5), chronological age must not be the sole criterion to develop public policies for older adults, and thus, our findings also pose implications for policymakers. Older adults who are institutionalized and/or participate in community centers (such as a significant proportion of participants in Profiles 1 and 2) require resources and interventions that help maintain social relationships and pleasurable experiences. The dimension of food can play a substantial role in this regard. Our findings suggest that food-related life should not only focus on the nutritional aspect, but also on the hedonic aspect, which includes eating in company, a pleasant meal atmosphere, and having access to food that is both healthy and tasty. Policies can support long-term stay institutions and community centers for older adults in the development and provision of meals that are healthy and palatable and that consider diverse physiological difficulties for food consumption (e.g., older adults' oral health and dietary restrictions). Policies can also provide older adults with benefits for purchasing healthy food, to promote healthier diets among those who live independently, regardless of their financial situation.

## 5. Conclusion

Using latent profile analysis, three latent profiles of older adults were identified based on their scores of life and food-related life satisfaction. Profile 1 comprised *older adults unsatisfied with their life, somewhat satisfied with their food-related life* (5.40% of the sample); Profile 2 included *older adults somewhat satisfied with their life, satisfied with their food-related life* (65.06%); and Profile 3 comprised *older adults extremely satisfied with their life and food-related life* (29.54%). Profiles significantly differed in terms of some eating habits, financial wellbeing, perceived social support from family, friends, and significant others, age, living situation (i.e., living in an ELEAM or a CAM), marital status, if older adults had an ever-confirmed COVID-19 infection previous to this study, and city of residence. The variables associated with belonging to the profile with higher levels of satisfaction with life and food-related life were living situation, city of residence, if the older adult had an ever-confirmed COVID-19 infection prior to this study, financial wellbeing, perceived social support from friends and significant others, diet quality, and age.

## Data availability statement

The raw data supporting the conclusions of this article will be made available by the authors, without undue reservation.

## Ethics statement

The studies were conducted in accordance with the local legislation and institutional requirements. Written informed consent for participation was required from the participants or the participants' legal guardians/next of kin.

## Author contributions

BSch conceived the study and wrote the first manuscript draft, approved the statistical analysis, and the final version of the manuscript. CA-B and ML guided the statistical analysis. LO, GL, and KB supervised data collection and made a critical analysis of the final version of the manuscript. All authors read and approved the final manuscript.
